# *In Vitro* and* Ex Vivo* Chemopreventive Action of* Mauritia flexuosa* Products

**DOI:** 10.1155/2018/2051279

**Published:** 2018-06-03

**Authors:** Joilane Alves Pereira-Freire, George Laylson da Silva Oliveira, Layana Karine Farias Lima, Carla Lorena Silva Ramos, Stella Regina Arcanjo-Medeiros, Ana Cristina Silva de Lima, Sabrina Almondes Teixeira, Guilherme Antônio Lopes de Oliveira, Nárcia Mariana Fonseca Nunes, Vivianne Rodrigues Amorim, Luciano da Silva Lopes, Larissa Araújo Rolim, Joaquim Soares da Costa-Júnior, Paulo Michel Pinheiro Ferreira

**Affiliations:** ^1^Department of Nutrition, Federal University of Piauí, 64607-670 Picos, Brazil; ^2^Postgraduate Programs in Pharmaceutical Sciences and Biotechnology, Federal University of Piauí, 64049-550 Teresina, Brazil; ^3^Department of Biology, Center for Higher Studies of Coelho Neto, State University of Maranhão, 65620-000 Coelho Neto, Brazil; ^4^Postgraduate Program in Biotechnology, Federal University of Ceará, 60020-181 Fortaleza, Brazil; ^5^Postgraduate Program in Foods and Nutrition, Federal University of Piauí, 64049-550 Teresina, Brazil; ^6^Department of Biophysics and Physiology, Laboratory of Experimental Cancerology, Federal University of Piauí, 64049-550 Teresina, Brazil; ^7^Department of Pharmaceutical Sciences, Federal University of Vale do São Francisco, 56304-205 Petrolina, Brazil; ^8^Federal Institute of Piauí, 64000-060 Teresina, Brazil

## Abstract

*Mauritia flexuosa* (Arecaceae), known as “Buriti,” is a Brazilian palm tree with high economic potential for local communities. Herein, we investigated the phytochemistry profile and antioxidant potential of* M. flexuosa* fruits and determined the bioaccessibility of phenolic compounds. Peels revealed upper values for phenols, flavonoids, carotenoids, tannins, and ascorbic acid when compared to the pulps and endocarps. All samples showed capacity to scavenger free radicals (0.5, 1.0, 2.0, 4.0, and 8.0 mg/mL) but peels presented higher scavenger action in all methods explored. Phenolic compounds identified by HPLC displayed reduced bioaccessibility after* in vitro* simulated gastrointestinal digestion for pulp (38.7%), peel (18.7%), and endocarp (22.3%) extracts (*P* < 0.05). Buriti fruits also protected rat blood cells against lysis induced by peroxyl radicals. We demonstrated the promising chemopreventive potentialities of* M. flexuosa* fruits and their by-products and peels with higher quantities of bioactive compounds and phenolic substances before and after* in vitro* bioaccessibility investigation. In Brazil, these parts are discarded or underused, mainly as feed for ruminant animals. Consequently, it is extremely important to explore nutritional characteristics of these by-products for human/livestock foods and to install biofriendly techniques and sustainable biotechnology handling of natural resources.

## 1. Introduction

Bioactive compounds have natural functions in plants such as sensory properties (color, aroma, flavor, and astringency) and defense against microorganisms and predators [1]. On the other hand, intake of vegetal nutrients has functional benefits for consumers and enables increasing supply for healthy foods. A diet rich in antioxidant compounds associated with endogenous enzymatic mechanisms can help to minimize the development of oxidative damage caused by free radicals (free electrons), mainly reactive oxygen (ROS)/nitrogen (RNS)/sulfur (RSS)/and chlorine species, since these unstable molecules are consequence of normal and/or unbalanced metabolic activities and studies have demonstrated epidemiological and biological correlations with chronic or nonchronic diseases such as hypercholesterolemia, atherosclerosis, hypertension, ischemia-reperfusion injury, inflammation, cystic fibrosis, diabetes, Parkinson's disease, Alzheimer, cancer, and aging process itself or premature aging [2–8].

In this context, plant species produce secondary metabolites belonging to different chemical groups such as alkaloids and cyanogenic glycosides and nonnitrogenous compounds, such as tannins, flavonoids, terpenes, and anthocyanins, which present antioxidant activity [9–12].

“Buriti,”* Mauritia flexuosa* L. f., belongs to the family Arecaceae, a palm tree widely distributed in South America, especially in the Amazon region and Brazilian Cerrado, where it has demonstrated high economic potential for the biotechnology development based on the sustainability of natural resources. In the Brazilian food industry, the peel and endocarp are commonly discarded or underutilized for the preparation of candies, ice creams, juices, jams, porridges, and/or oils [13]. Additionally, some studies have emphasized pharmacological potentialities of the* M. flexuosa* parts, such as antimicrobial [14–16], antitumor [16], hypolipemiant [17], hypoglycemiant [18], and healing activities [19].

For exotic and underexploited plants, in particular, there is little and shallow knowledge about key interfering factors in the biological significance of foods on human health, intake of nutrients, and their bioaccessibility/bioavailability throughout the gastrointestinal tract [20, 21]. In this perspective, the development of studies on the use of regional and tropical fruits should be encouraged, advancing about the knowledge and exploring the use of fresh fruits for Research and Development (R&D) of novel products [22, 23]. Herein, we investigated the phytochemistry profile and antioxidant potential of* M. flexuosa* fruits and determined the bioaccessibility of phenolic compounds using* in vitro* simulated gastrointestinal digestion.

## 2. Materials and Methods

### 2.1. Chemical Reagents

Chemical reagents 2,2-diphenyl-1-picrylhydrazyl (DPPH^*∙*^), 2,20-azino-bis(3-ethylbenzothiazoline-6-sulfonic acid) (ABTS^*∙*+^), thiobarbituric acid, trichloroacetic acid, ferric chloride, potassium ferricyanide, dihydrochloride 2,2′-azobis(2-amidinopropane) dihydrochloride (AAPH), sodium nitroprusside (SNP), Triton X-100, Folin-Ciocalteu, sodium carbonate, gallic acid, aluminum chloride, quercetin, *β*-carotene, potassium iodide, and potassium persulfate were obtained from Sigma-Aldrich Co. (St. Louis, MO, USA).

### 2.2. Plant Material: Origin and Preparation

**Figure 1:**
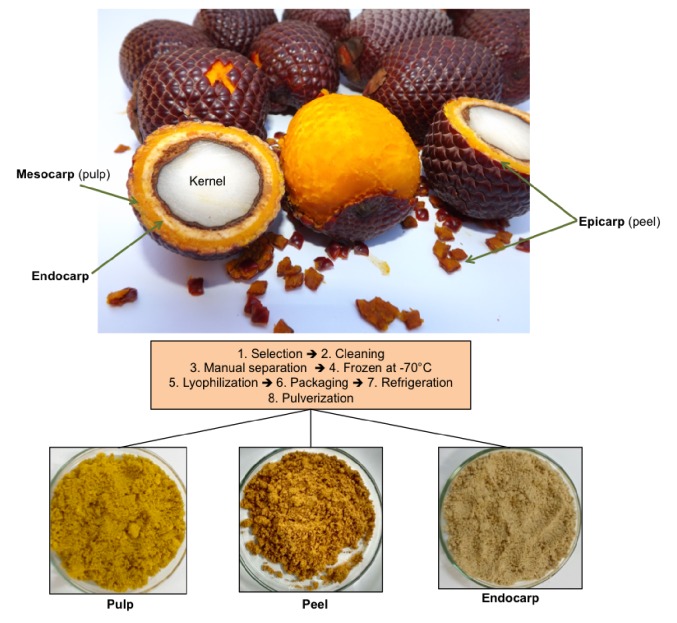
Preparation of* Mauritia flexuosa* fruits: lyophilization, pulverization, and stocking preceded phytochemical and biological analysis.

A sample of* Mauritia flexuosa *was deposited in the Graziela Barroso Herbarium at Federal University of Piauí (UFPI) (voucher specimen #30567). About 300 fruits were collected in Água Branca, Piauí, Brazil, in December 2014 (latitude: 5°54′50.5′′S; longitude: 42°38′03.4′′W) and taken to the Federal Institute of Piauí, Teresina, Brazil. Fruits were selected regarding sanity and same maturation stage and cleaned in water containing 25 ppm of commercial sodium hypochlorite. These fruits presented an elongated oval shape surrounded by the epicarp (peel) of reddish brown color, mesocarp (pulp), orange, and endocarp with a white or yellowish spongy tissue [24]. Subsequently, fruits were separated in pulp, peel, and endocarp. These parts were frozen separately at −70°C. For the lyophilization process, stainless steel tray of lyophilizer model L101 (Liotop, São Carlos, Brazil) was used. Lyophilization conditions (temperature: 40°C; vacuum pressure: <500 mmHg; lyophilization rate: 1 m/h) were well controlled during 72 h [25]. After such process, fruits were packaged in plastic bags under refrigeration at 4°C before process for preparation of powder samples using rotor mill (0.08 mm) (Figure 1).

### 2.3. Content of Phenols, Flavonoids, Carotenoids, Tannins, and Ascorbic Acid

Pulverized pulp, peel, and endocarp samples were submitted to extraction of bioactive compounds with methanol. Samples were mixed with mortar and pestle for 10 min (1 : 10; sample/solvent) until reaching uniform consistency. Methanol extracts were stored at 4°C for 2 days up to quantification of bioactive compounds (phenols, flavonoids, carotenoids, and tannins) and antioxidant activity, respectively. All analyses of bioactive compounds were carried out in quintuplicate.

#### 2.3.1. Total Phenolics

The total phenolic content was determined with Folin-Ciocalteu reagent according to [3], with some modifications. For 1 mL of pulp, peel, and endocarp methanol solution (10 mg/mL), 1 mL of Folin-Ciocalteu reagent (1 : 4) and 1 mL of 15% sodium carbonate (Na_2_CO_3_) were added and the final volume was filled with distilled water to 10 mL. The mixture was maintained for 2 h and centrifuged at 4000 rpm during 4 min. The supernatant was measured at 750 nm. Stock solution without fruit parts was used as negative control. Results were expressed as mg of gallic acid equivalents per 100 g of sample (mg GAE/100 g sample) and a gallic acid calibration curve was determined (0.9497*x*  *y* = − 0.0527; *r*^2^ = 0.999).

#### 2.3.2. Total Flavonoids

The content of total flavonoids was determined based on the formation of the flavonoid-aluminum complex, according to [3] with some modifications. For 1 mL of pulp, peel, and endocarp methanol solution (10 mg/mL), 1 mL of 20% aluminum chloride and 100 *μ*L of 50% acetic acid were added. The mixture was maintained for 30 min and centrifuged at 4000 rpm during 4 min. The supernatant was measured at 420 nm. Results were expressed as mg of quercetin equivalent per 100 g of sample (mg EQE/100 g sample) and a quercetin calibration curve was prepared (*y* = 0.0136*x*  −  0.0422; *r*^2^ = 0.999).

#### 2.3.3. Total Carotenoids

Total carotenoids were determined according to [26] with some modifications. A total of 0.1 g of pulp, peel, and endocarp diluted in 10 mL of acetone : hexane solution (4 : 6) was stirred for 10 min at room temperature (400 rpm) and centrifuged for 4 min at 4000 rpm. Reading was performed at 450 nm and the results were expressed as mg of *β*-carotene equivalent per 100 g of sample (mg *β*CTE/100 g sample). A *β*-carotene calibration curve was prepared (*y* = 0.3099*x*  −  0.341; *r*^2^ = 0.991).

#### 2.3.4. Condensed Tannins

The content of condensed tannins was determined using the methodology of vanillin [27]. To the methanol solution containing 1 mL of pulp, peel, and endocarp (10 mg/mL), 3 mL of 2% vanillin prepared with sulfuric acid (70%) was added. Subsequently, the reaction mixture was performed in water bath at 20°C for 15 min. Samples were centrifuged for 4 min at 4000 rpm and reading was carried out in digital spectrophotometer at 500 nm. Results were expressed as milligrams of catechin equivalents per gram of sample (mg CTQ/100 g sample). A catechin calibration curve was performed (*y* = 0.008*x* + 0.096; *r*^2^ = 0.999).

#### 2.3.5. Hydrolysable Tannins

The hydrolysable tannin concentration was determined using potassium iodide according to [28]. One milliliter of saturated potassium iodide solution was added to the methanol solution containing 3 mL of pulp, peel, and endocarp (10 mg/mL) and allowed to rest at room temperature for 40 min and centrifuged for 4 minutes at 4000 rpm and the absorbance was measured at 550 nm. Results were expressed as mg of tannic acid equivalents per gram of sample (mg ACT/100 g sample) and a tannic acid calibration curve (0.0122*x* + *y* = 0.26; *r*^2^ = 0.981) was performed.

#### 2.3.6. Ascorbic Acid 

Ascorbic acid content was determined using the titrimetric* Tillmans*' method. We used a solution of oxalic acid as a solvent to substitute metaphosphoric acid. Twenty milliliters was mixed with 80 mL of 1% oxalic acid and 10 mL of such solution was titrated with Tillmans reagent, using 2,6-dichlorophenolindophenol. Results were calculated based on a standard solution of ascorbic acid and expressed in mg/100 mL.

### 2.4. In Vitro Quantification of Total Phenolics after Simulated Gastrointestinal Digestion

The digestion was performed using simulated gastric (pepsin solubilized with 0.1 mol/L HCl) and intestinal fluids (pancreatin-bile salts solubilized with 0.1 mol/L NaHCO_3_), which were prepared according to [29]. We added 1 mL of pulp, peel, and endocarp methanol solution (10 mg/mL) to 100 mL of 0.01 mol/L HCl, and pH was adjusted to 2 with 2 mol/L HCl solution. Equal quantity of phenols was used as positive control (10 mg/mL). Afterwards, 3.2 mL of pepsin was added, maintaining samples under stirring at 37°C for 2 h to simulate food digestion in the stomach. Then, to simulate the pH found in human intestines, titration was carried out with 0.5 mol/L NaOH to obtain pH 7.5. Subsequently, a dialysis process was performed for 2 h (dialysis membrane with 33 × 21 mm, molecular weight of 12.000 to 16.000, and porosity of 25 angstroms, Inlab, Brazil) with 0.1 mol/L NaHCO_3_ equivalent to titratable acidity. After pH adjustment, dialysis membranes were added and the solution was stirred in water bath at 37°C/30 min. Then, 5 mL of pancreatin-bile salts solution was added and the mixture was stirred again for additional 2 h to mimic food digestion in the intestine. Finally, the membrane content (dialysate) was removed and samples were stored at 20°C until analysis.

Finally, dialyzed material was analyzed to determine total phenolics [3]. Results were expressed as mg gallic acid/100 g sample. The bioaccessible percentage was calculated according to [20]: % bioaccessible = 100 × (DPC/CPC), where F is the content of dialyzable phenolic compounds (mg gallic acid/100 g sample) and G corresponds to the content of phenolic compounds in the sample (mg gallic acid/100 g sample).

### 2.5. In Vitro Antioxidant Capacity

For* in vitro* antioxidant evaluation, the antioxidant capacity of the samples was assayed against 1,1-diphenyl-2-picrylhydrazyl [DPPH^*∙*^] [30], 2,2′-azino-bis(3-ethylbenzothiazoline-6-sulphonic acid [ABTS^*∙*+^] [31], reducing potential [Fe^3+^/Fe^2+^] [32], lipid peroxidation [thiobarbituric acid reactive substances (TBARS) removal [33, 34], and nitrite content [nitrite production induced by sodium nitroprusside [35, 36]. Aqueous stock solutions of the samples (pulp, peel, and endocarp: 0.5, 1.0, 2.0, 4.0, and 8.0 mg/mL), DPPH^*∙*^ (40 *μ*g/mL), ABTS^*∙*+^ (7 mM), 1% potassium ferricyanide, sodium nitroprusside (10 mM), and 0.67% thiobarbituric acid, were prepared. Trolox (0.5 mg/mL) was used as positive standard.

Values of 50% effective concentration (EC_50_) for Buriti extracts were spectrophotometrically quantified (T80+ UV/VIS Spectrometer, PG Instruments Ltd.®, Leicestershire, UK) at 515 nm for DPPH^*∙*^, 734 nm for ABTS^*∙*+^, 700 nm for potassium ferricyanide, 532 nm for TBARS (thiobarbituric acid reactive substances), and 540 nm for nitrite radicals 30 min after the reaction started. Antioxidant evaluation was performed in triplicate from two independent experiments and absorbance values were converted to the inhibition percentage (I) of radicals using the equation of [37]: (%) = [(absorbance of negative control − absorbance of sample) × 100]/absorbance of negative control, where absorbance of negative control is, for example, the initial absorbance for DPPH^*∙*^ solution and absorbance of sample is the absorbance for reaction mixture (DPPH^*∙*^ and sample).

### 2.6. Ex Vivo Analysis on Murine Erythrocytes

All procedures were approved by the Committee on Animal Research at UFC (#054/2014) and they are in accordance with Brazilian (COBEA,* Colégio Brasileiro de Experimentação Animal*) and international guidelines on the care and use of experimental animals (Directive 2010/63/EU of the European Parliament and of the Council).

Blood was collected from retroorbital plexus of anesthetized female* Wistar* rats (180–220 g) with ketamine (90 mg/kg, i.p.) and xylazine (10 mg/kg, i.p.). Blood was mixed with 0.85% NaCl solution containing 10 mM CaCl_2_ and submitted to three centrifugations (2000 rpm/5 min). Erythrocytes were suspended in NaCl to obtain a cell suspension (10%). Hemolytic investigations were performed in 96-well plates following the method described by [38].

#### 2.6.1. Hemolytic Capacity Determination

Each well received 50 *μ*L of 0.85% NaCl. The first well was the negative control that contained only the vehicle (PBS), and in the second well 50 *μ*L of test substance that was diluted in half was added. The extracts were tested at concentrations ranging from 0.5 to 8 g/mL. The last well received 50 *μ*L of 0.2% Triton X-100 (in 0.85% saline) to obtain 100% hemolysis. Then, each well received 50 *μ*L of a 10% suspension of mice erythrocytes in 0.85% saline containing 10 mM CaCl_2_. After incubation at room temperature for 2 h, cells were centrifuged, the supernatant was removed, and the liberated hemoglobin was measured spectroscopically as absorbance at 540 nm. For comparison, a solution of 0.5 mg/mL Triton X-100 was used as positive control.

#### 2.6.2. Antioxidant Capacity against Oxidative Hemolysis

The antioxidant capacity against oxidative hemolysis was performed by inhibition of oxidative hemolysis induced by peroxyl radicals generated following thermal decomposition of 2,2′-azobis(2-amidinopropane) dihydrochloride (AAPH) in method described by [39] with some modifications. Briefly, aliquots of pulp, peel, and endocarp aqueous extracts (0.5 to 8 mg/mL) were mixed with 30 *μ*L of 10% erythrocyte suspension and 50 *μ*L of AAPH (200 mM in PBS, pH 7.4) in 96-well plates. The reaction mixture was incubated for 120 minutes at 37°C. Afterwards, the reaction mixture was diluted with 240 *μ*L of PBS and centrifuged at 2000 rpm for 5 min and the liberated hemoglobin was measured spectroscopically as absorbance at 540 nm. Results were expressed as percentage inhibition of hemolysis compared to the complete hemolysis of erythrocyte suspensions induced by AAPH. Liberated hemoglobin was measured spectroscopically as absorbance at 540 nm. The inhibition of erythrocyte hemolysis was calculated as (1 − *A*_sample_/*A*_control_) × 100%. Trolox (0.5 mg/mL) was used as positive standard.

### 2.7. Chromatographic Analysis

For chromatographic analysis, methanol extracts of pulp, peel, and endocarp were used. Mobile phases were represented by solvents A–C using three pumps associated with the chromatograph (Shimadzu® liquid chromatograph with a diode array detector, Japan: solvent A, 0.1% trifluoroacetic acid in acetonitrile; solvent B, 0.1% trifluoroacetic acid in HPLC grade water; solvent C, 100% methanol). A TSK-GEL Super-ODS (Supelco) column was used. The effluent was monitored at 250 and 330 nm. Flow rate was fixed at 1.0 mL/min, and column temperature was maintained at 37°C throughout the test. Initially, the solvent was represented by 100% solvent B, but a linear gradient was used to increase solvent A from 0 to 10% within 7 min. Its composition was maintained at an isocratic flow for 3 min. Then, solvent A increased from 10 to 40% during 20 min. Such composition was maintained for additional 2 min and returned to the initial condition in 3 min. A volume of 20 *μ*L for the standards substances and samples was injected for each HPLC analysis.

### 2.8. Statistical Analysis

Data were presented as mean ± standard error of the mean (SEM) and compared by one-way analysis of variance (ANOVA) followed by Student-Newman-Keuls test using GraphPad Prism® software 5.0 (San Diego, CA, USA). EC_50_ values were calculated by nonlinear regression (95%). Statistical correlation among experimental data was performed using the Pearson coefficient (*r*) and results were statistically significant when *P* < 0.05.

## 3. Results

### 3.1. Bioactive Compounds and Bioaccessibility

The screening of bioactive compounds in* M. flexuosa *fruit is described in Table 1. Peel revealed the highest values for phenols (1288.0 ± 10.4 mg GAE/100 g), flavonoids (339.4 ± 3.9 mg EQE/100 g), carotenoids (88.3 ± 0.3 mg *β*CTE/100 g), tannins (hydrolysable: 56.2 ± 0.4 mg ACT/100 g; condensed: 118.3 ± 2.1 mg CTQ/100 g), and ascorbic acid (5.9 ± 0.2 mg/100 mL) when compared to the pulp and endocarp (*P* < 0.05).

The correlation of chromatographic peaks was achieved by comparison of experimental retention times (*t*_*R*_) with reference standards (Table 2). All chromatographic analyses were carried out in triplicate and revealed phenolic compounds (protocatechuic acid, quercetin, apigenin, catechin, and epicatechin) with the following *t*_*R*_: 16.3, 33.6, 41.7, 53.6, and 49.3 minutes, respectively.

Subsequently, we analyzed the quantity of phenolic compounds before and after* in vitro* simulated gastrointestinal digestion for pulp, peel, and endocarp (Table 3). All samples (pulp, peel, and endocarp) displayed reduction in bioaccessibility after* in vitro* digestion of 38.7, 18.7, and 22.3%, respectively (*P* < 0.05).

### 3.2. In Vitro Antioxidant Capacity

**Figure 2:**
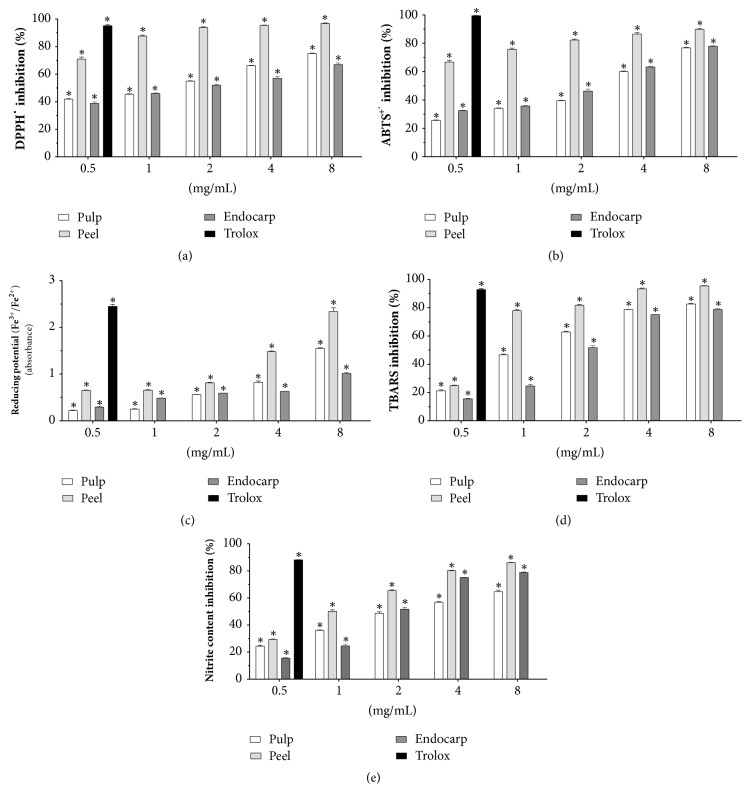
Effects of lyophilized fruits (pulp, peel, and endocarp) from* Mauritia flexuosa* (0.5, 1, 2, 4, and 8 mg/mL) on the removal of (a) 1,1-diphenyl-2-picrylhydrazyl (DPPH^*∙*^), (b) 2,20-azino-bis(3-ethylbenzothiazoline-6-sulfonic acid) (ABTS^*∙*+^), (c) reducing potential (Fe^3+^/Fe^2^), (d) reactive substances to thiobarbituric acid [TBARS levels induced by 2,2′-azo-bis (2-methylpropionamidine]) dihydrochloride, AAPH), and (e) nitrite content (induced by sodium nitroprusside). Trolox (0.5 mg/mL) was used as positive standard. Results are expressed as mean ± standard error of measurement (SEM) from two independent experiments in triplicate. Negative control was treated with the solution used for diluting the tested substance. With exception of reducing potential, absorbance values were converted to inhibition (*I*) percentage of radicals: *I* (%) = [(absorbance of negative control − absorbance of sample) × 100]/absorbance of negative control. ^*∗*^*P* < 0.05 compared to negative control by ANOVA followed by Student-Newman-Keuls test.

In this step, we carried out quantification of the antioxidant capacity of Buriti samples (pulp, peel, and endocarp) at concentrations of 0.5, 1, 2, 4, and 8 mg/mL. This capacity is described as free radical inhibition (Figure 2).

For all parameters and samples, we determined EC_50_ values: 1.6 ± 0.1, 0.1 ± 0.1, and 1.5 ± 0.1 mg/mL (DPPH^*∙*^); 2.3 ± 0.1, 0.1 ± 0.1, and 1.9 ± 0.1 mg/mL (ABTS^*∙*+^); 2.1 ± 0.3, 1.2 ± 0.1, and 1.9 ± 0.4 mg/mL (potassium ferricyanide); 1.6 ± 0.2, 0.7 ± 0.1, and 2.3 ± 0.2 mg/mL (TBARS); and 2.6 ± 0.1, 1.1 ± 0.1, and 6.4 ± 0.14 mg/mL (nitrite content) for pulp, peel, and endocarp, respectively. Trolox (0.5 mg/mL), the positive standard, showed free radical inhibition capacity upper to 90% for the antioxidant assessments (Figure 2). Then, all samples showed growing capacity in a concentration-dependent manner to scavenger free radicals, but it is important to note that peels' samples presented a higher scavenger capacity in all methods explored (*P* < 0.05).

### 3.3. Antioxidant Capacity against Oxidative Hemolysis

Firstly, we analyzed the capacity of the samples to cause hemolysis. None of the extracts induced lysis of rat erythrocytes even 8.0 mg/mL. On the other hand, Triton X-100, used as positive control, caused 100% hemolysis.

**Figure 3:**
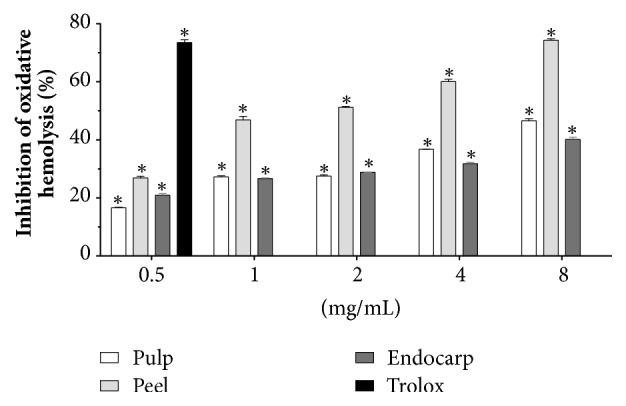
Protection against oxidative hemolysis induced by peroxyl radicals generated following thermal decomposition of 2,2′-azobis(2-amidinopropane) dihydrochloride (AAPH) by lyophilized fruits (pulp, peel, and endocarp) from* Mauritia flexuosa* (0.5, 1, 2, 4, and 8 mg/mL). Trolox (0.5 mg/mL) was used as positive standard. Results are expressed as mean ± standard error of measurement (SEM) from two independent experiments in triplicate. Negative control was treated with the solution used for diluting the tested substance. ^*∗*^*P* < 0.05 compared to control by ANOVA followed by Student-Newman-Keuls test.

Based on these promising findings (scavenger of free radicals and absence for cellular lysis), we evaluated the antioxidant capacity against oxidative hemolysis induced by AAPH (100% hemolysis). Once again, all concentrations used (0.5, 1.0, 2.0, 4.0, and 8.0 mg/mL) were able to protect blood cells when compared to positive control exposed to peroxyl radicals generated following thermal decomposition of AAPH as follows: pulp (15.0 ± 1.1, 26.9 ± 0.7, 27.6 ± 0.4, 36.8 ± 0.1, and 49.3 ± 2.7%), peel (26.9 ± 0.6, 46.9 ± 1.2, 51.2 ± 0.3, 60.1 ± 0.8, and 74.3 ± 0.5%), and endocarp (19.6 ± 1.7, 25.7 ± 0.9, 28.5 ± 0.3, 31.8 ± 0.5, and 40.2 ± 0.7%), respectively (Figure 3). Trolox showed an antioxidant perceptual protection of 73.2 ± 0.5%. EC_50_ values were 7.7 ± 0.4, 1.8 ± 0.1, and 11.4 ± 0.5 mg/mL for pulp, peel, and endocarp, respectively.

Pearson's correlation, a measure of the strength of linear relationship between two variables, revealed a positive relationship between bioactive compounds (total phenol, total flavonoids, total carotenoids, and condensed and hydrolysable tannins) and antioxidant capacity (*r* > 0.881; *P* < 0.05) and bioactive compounds and protection against oxidative hemolysis (*r* > 0.907; *P* < 0.05) (Table 4). On the other hand, Pearson's correlation did not show association between antioxidant activity against TBARS and presence of bioactive compounds for most correlations analyzed (*P* > 0.05).

## 4. Discussion

Since oxidative damage contributes significantly to pathologies, herein, we performed different biochemical methods to support the antioxidant and functional action of* M. flexuosa *fruits.

Peels from* M. flexuosa *fruits presented highest values of bioactive compounds when compared to the pulp and endocarps. Previously, studies demonstrated that pulp extracts from Amazon Buriti have mainly quinic acid, caffeic acid, chlorogenic acid, ferulic acid, p-Coumaric acid, protocatechuic acid, catechin, epicatechin, luteolin, apigenin, myricetin, kaempferol, and quercetin, some of them also found in lower concentrations [18]. Moreover, as confirmed here, Buriti seems to be an excellent source of carotenoids (44600 *μ*g/100 g), especially *α*- and *β*-carotene and* cis*- and* trans*-*ᵞ*-carotene [40–43], which are normally found in carrots and are considered the most known and accepted source by consumers, justifying its use to treat hypovitaminosis A.

Our results presented differences per 100 g of dry material, since Buriti samples were collected under natural conditions from Cerrado Brazilian (a type of savanna) and most studies presented outcomes with fruits from Amazon region. These findings are explained by differences in biome conditions. Amazon is hot and humid, while Cerrado presents a dryer climate. Besides, the Cerrado soil is more acid and rich in aluminum salts, which will probably generate higher oxidative stress for the plants. They react, producing antioxidant agents [41].

Polyphenol substances with high* in vitro* antioxidant activity do not necessarily have similar actions after gastrointestinal process and absorption [20, 44]. Therefore, we verified the bioaccessibility of phenolic compounds from pulp, peel, and endocarp methanol extracts. For this, we used an* in vitro* method that has recently gained much attention because it simulates the process of gastrointestinal digestion, enabling studying changes that occur in the diet components during gastric and intestinal digestion. Moreover,* in vitro* techniques have the advantage to substitute animals and are time-efficient and cost-effective and require less manpower [21, 23, 44, 45]. Interestingly,* M. flexuosa* methanol extracts showed reduction of bioaccessible polyphenols after digestion simulation ranging from 18.7 (pulp) to 38.7% (peel).

It is important to note that only solubilized nutrients from the food matrix which are not destroyed during gastrointestinal digestion are bioaccessible and potentially bioavailable [22, 23]. Since dietary fiber components are not absorbed, they achieve the large intestine and provide the substrate for intestinal digestion. Soluble fibers are usually fermented quickly, while insoluble fibers are slowly or only partially fermented. The fermentation is carried out by anaerobic bacteria of the colon (e.g.,* Lactobacillus* and* Bifidobacterium *genera), leading to the production of lactic acid, short-chain fatty acids, and gas, events that can alter food components and their bioavailability [46]. Furthermore, the consumption of high quantities of phytates and oxalates can cause chelation of metal ions (e.g., calcium and zinc) and induce cholelithiasis [24].

Although* M. flexuosa* fruits have been associated with multiple nutritional properties that can be favorable to the human health, their fibers and polyphenols may link to macromolecular compounds that are not dialyzable or generate mineral complexes, further decreasing solubility and bioaccessibility of phenols [47, 48]. Furthermore, because dialysis process during* in vitro *gastrointestinal digestion separates bioactive substances, this can interfere with biological activity and quantity of phenolic compounds, which may work more efficiently together rather than individually as synergists to reduce free radicals [49].

Investigators working with cashew fruits from* Anacardium occidentale* L., another typical natural delight from Brazilian Northeast known as “caju,” “acajuíba,” and “açajaíba,” but more popular, accepted, studied, and economically exploited than* M. flexuosa*, also showed a considerable loss of phenolic compounds in cashew apple juice and cashew apple fiber after bioaccessibility tests, mainly due to the type of food matrix elements, and this often alters absorption of phenolic compounds [44].

*In vitro* antioxidant activity is mainly based on chemical assays that assess the ability of a substance to reduce the concentration of free radicals in a specific reaction medium [50, 51]. Then, we performed methods to determine the* in vitro* scavenging actions.

Firstly, we used the DPPH method, since it is a rapid, simple, accurate, and inexpensive assay for measuring the ability of different compounds to act as free radical scavengers or hydrogen donors and to evaluate the antioxidant activity of foods and beverages independent of sample polarity [11, 52]. In the ABTS test, 2,2′-azino-bis (3-ethylbenzthiazoline-6-acid) (ABTS) is converted into its radical (ABTS^*∙*+^) by addition of sodium persulphate and is reactive towards most antioxidants. Since it is not affected by ionic strength, it can be used to determine both hydrophilic and hydrophobic antioxidant capacities [10]. The total antioxidant activity was also measured by the ferric reducing antioxidant power assay. Flavonoids and phenolic acids presented in the medicinal plants exhibit strong antioxidant activity, which is depending on their potential to form the complex with metal atoms, particularly iron and copper. This method is based on the principle of increase in the absorbance of the reaction mixtures [32]. Subsequently, lipid peroxidation was determined by TBARS removal. Since polyunsaturated fatty acids are easy targets for oxidants, and the process of lipid peroxidation is, once initiated, a self-sustaining free radical chain process, the accumulation of lipid peroxidation products provides the most common biochemical marker of oxidative stress [33, 34]. Finally, nitrite ion technique was carried out based on the decomposition of sodium nitroprusside in nitric oxide at physiological pH, under aerobic conditions, which produces nitrites [35, 36]. It was important to perform the evaluation of samples against RNS, since these radicals may cause damage to biological components such as the aromatic amino acid tyrosine and DNA bases, particularly in guanines, by nitration or hydroxylation [51].

Buriti samples presented antioxidant capacity, and peel extracts were more active scavengers. References [14] also demonstrated antioxidant potential in leaves (iron reduction test) and fruit pulps (DPPH method) from* Mauritia flexuosa*. Differences in the antioxidant action found are probably associated with distinctive concentrations of the chemical constituents in each part of the plant, mainly flavonoids and anthocyanins [53]. So, there is a huge possibility that this effect repeats in Buriti fruits in different Brazilian regions, once* M. flexuosa* in the “Cerrado” biome is exposed to a higher incidence of sunlight in a soil of dry climate [54]. It is supposed that climate conditions interfere even in the constitution of the general parts, with average values of 22.1–25.1, 11–24.2, 21.0, and 32.6–63.9% for peel, pulp, endocarp, and seed, respectively [25, 55].

Typically, phenols and carotenoids are found in higher concentrations in peels due to their pigmentation, regulation of enzymatic activity, and protection against sunlight and pathogenic microorganisms [1, 56]. So, we noted superior presence of phenolic compounds (57.0 and 53.6%), flavonoids (22.1 and 57.2%), tannins (hydrolysable: 15.7 and 99.8%; condensed: 41.1 and 69.1%), and ascorbic acid levels in peels when compared to pulp and endocarp, respectively, which improved antioxidant activity in peels, respectively. Taking into consideration the fact that the Dietary Reference Intake (DRI) of ascorbic acid for adults is 45 mg/day [57], one cup with 200 mL of peel extract from* M. flexuosa* fruits (11.7 mg/mL) would correspond to 26% of the RDI, while consumption of pulp would reach 19.1%. Anyway, it is important to note that vitamin C is converted to oxalate when it is present in higher concentrations [24].

For* M. flexuosa* fruit, protection by antioxidant compounds is required and could be a reason for the higher concentration of bioactive compounds found in the peel than in pulp and endocarp. Using Pearson's correlation, we found a good correlation index among bioactive compounds and antioxidant capacity for pulp, peel, and endocarp from* Mauritia flexuosa*, which supports the suggestion that protection against oxidative hemolysis is directly associated with levels of bioactive substances.

Since vegetal extracts are rich in different classes of compounds that can attack or interact with cellular membranes, hemolysis assay is frequently used to test materials, compounds, or mixture of compounds at defined pHs that mimic extracellular environments. So, the evaluation of membrane stability during exposure to phytotherapeutic products must be routinely considered in their evaluation, since the consumption of these products is increasing globally and may constitute a serious public health problem. So, membrane stability represents the capacity of this biological complex to maintain its structure under chaotropic conditions such as hypotonicity, pH extremes, heat, and the presence of solutes (such as ethanol, urea, and guanidine) and oxidative stress [38, 58–60]. When submitted to the cell assays, none of the Buriti samples caused lysis of erythrocytes and reversed hemolysis induced by peroxyl radicals and, once again, better results were found with peel extracts.

The antihemolytic action described for fruit extracts from* M. flexuosa* may be associated with an osmotic stabilization of erythrocytes. It is possible that the exacerbation of Van der Waals contacts inside the lipid bilayer could be a source of membrane stabilization, though such membrane protection is normally related to the prevention of lipoperoxidation triggered by secondary metabolites such as flavonoids and phenols that can be incorporated into erythrocyte membranes [39, 58, 61]. Indeed, there is a strong correlation between thiobarbituric acid-reactive substances (TBARS) as a marker of lipid peroxidation and products that protect cells against oxidative damage [50]. Such protection can explain, at least in part, some folk uses and pharmacological properties of these fruits, such as protective effects against cognitive impairment [24, 62], antiplatelet, antithrombotic [63], lowering cholesterol [43, 64], and healing [19, 41] activities.

## 5. Conclusions

In summary, the antioxidant analysis of* M. flexuosa* fruits and their by-products showed promising chemopreventive potentialities, and peels demonstrated higher quantities of bioactive compounds and phenolic substances before and after* in vitro* bioaccessibility investigation. Since the processing of* M. flexuosa* fruits generates a large quantity of agricultural residues such as peels, endocarps, and seeds, most of them are commonly discarded or are used as feed for ruminant animals only, especially after production of sweets and oil extraction. Consequently, it is extremely important to explore the nutritional characteristics of these by-products for human/livestock foods and to install biofriendly techniques and sustainable biotechnology handling of natural resources. For Brazilian local communities, it is really important to reuse such residues, especially for people from poor regions, as a way to give better opportunities and improve quality of life.

## Figures and Tables

**Table 1:** Quantification of phenols, flavonoids, carotenoids, condensed tannins, and hydrolysable tannins in the lyophilized methanolic extracts of pulp, peel, and endocarp from *Mauritia flexuosa* fruits.

Class of compounds	Pulp	Peel	Endocarp
Total phenols (mg GAE/100 g)	553.5 ± 7.7^b^	1288.0 ± 10.4^a,c^	597.1 ± 6.5^b^
Total flavonoids (mg EQE/100 g)	264.4 ± 2.1^b,c^	339.4 ± 3.9^a,c^	145.4 ± 10.2^a,b^
Total carotenoids (mg *β*CTE/100 g)	58.9 ± 0.1^b,c^	88.3 ± 0.3^a,c^	19.1 ± 0.2^a,b^
Hydrolysable tannins (mg ACT/100 g)	47.4 ± 0.3^b,c^	56.2 ± 0.4^a,c^	0.1 ± 0.02^a,b^
Condensed tannins (mg CTQ/100 g)	69.6 ± 1.8^b,c^	118.3 ± 2.1^a,c^	36.5 ± 1.4^a,b^
Ascorbic acid (mg/100 mL)	4.3 ± 1.3^c^	5.9 ± 0.2^c^	2.5 ± 0.3^a,b^

Data were presented as mean ± standard error of the mean (SEM). ^a^*P* < 0.05 compared to pulp; ^b^*P* < 0.05 compared to peel; ^c^*P* < 0.05 compared to endocarp by ANOVA followed by Student-Newman-Keuls test.

**Table 2:** Identification of compounds by high-performance liquid chromatography (HPLC) in *Mauritia flexuosa *samples.

IUPAC Name Chemical Name	Chemical structures	Class	Retention time (min)	Sample
3,4-Dihydroxybenzoic acid (protocatechuic acid)	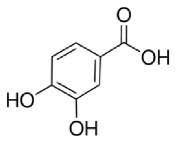	Phenol	16.3	Pulp

2-(3,4-dihydroxyphenyl)-3,5,7-trihydroxychromen-4-one *(quercetin)*	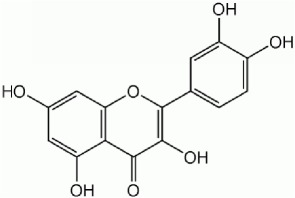	Flavonoid	33.6	Pulp

4′,5,7-Trihydroxyflavone *(apigenin)*	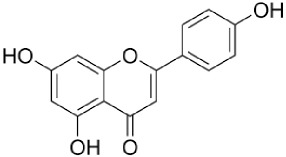	Flavonoid	41.7	Pulp Endocarp

(−)-*trans*-3,3′,4′,5,7-pentahydroxyflavane, (2S,3R)-2-(3,4-dihydroxyphenyl)-3,4-dihydro-1(2H)-benzopyran-3,5,7-triol (catechin)	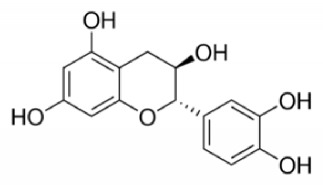	Condensed tannin	53.6	Endocarp Peel Pulp

(−)-*cis*-3,3′,4′,5,7-pentahydroxyflavane, (2*R*,3*R*)-2-(3,4-dihydroxyphenyl)-3,4-dihydro-1(2*H*)-benzopyran-3,5,7-triol (epicatechin)	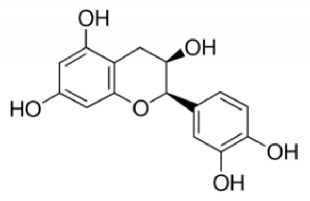	Condensed tannin	48.3	Peel

**Table 3:** Contents of phenolic compounds in the lyophilized methanolic extracts of pulp, peel, and endocarp from *Mauritia flexuosa* fruits before and after simulated gastrointestinal digestion.

Sample	Bioaccessibility before *in vitro* digestion (mg/L)	Bioaccessibility after *in vitro* digestion (mg/L)	Reduction (%)
Pulp	553.5 ± 7.7	102.2 ± 0.4^*∗*^	18.7
Peel	1288.0 ± 10.4	498.5 ± 13.9^*∗*^	38.7
Endocarp	597.1 ± 6.5	133.4 ± 7.8^*∗*^	22.3

^*∗*^ *P* < 0.05 compared to bioaccessibility before *in vitro* digestionby ANOVA followed by Student-Newman-Keuls test.

**Table 4:** Analysis of Pearson's correlation among bioactive compounds and antioxidant capacity in samples of pulp, peel, and endocarp from *Mauritia flexuosa*.

Class of compounds	DPPH^*∙*^	ABTS^*∙*+^	Reducing potential	TBARS	Nitrite content	Oxidative hemolysis
Pulp						
Total phenols	0.956^*∗*^	0.978^*∗*^	0.978^*∗*^	0.867	0.931^*∗*^	0.954^*∗*^
Total flavonoids	0.957^*∗*^	0.979^*∗*^	0.978^*∗*^	0.869	0.933^*∗*^	0.956^*∗*^
Total carotenoids	0.951^*∗*^	0.974^*∗*^	0.975^*∗*^	0.859	0.926^*∗*^	0.951^*∗*^
Condensed tannins	0.955^*∗*^	0.977^*∗*^	0.978^*∗*^	0.866	0.930^*∗*^	0.954^*∗*^
Hydrolysable tannins	0.923^*∗*^	0.953^*∗*^	0.956^*∗*^	0.822	0.898^*∗*^	0.935^*∗*^

Peel						
Total phenols	0.681	0.847	0.928^*∗*^	0.749	0.854	0.907^*∗*^
Total flavonoids	0.956^*∗*^	0.978^*∗*^	0.978^*∗*^	0.867	0.931^*∗*^	0.954^*∗*^
Total carotenoids	0.966^*∗*^	0.984^*∗*^	0.983^*∗*^	0.881^*∗*^	0.941^*∗*^	0.959^*∗*^
Condensed tannins	0.963^*∗*^	0.982^*∗*^	0.982^*∗*^	0.876	0.937^*∗*^	0.957^*∗*^
Hydrolysable tannins	0.972^*∗*^	0.988^*∗*^	0.987^*∗*^	0.890^*∗*^	0.947^*∗*^	0.961^*∗*^

Endocarp						
Total phenols	0.682	0.848	0.930^*∗*^	0.751	0.854	0.907^*∗*^
Total flavonoids	0.951^*∗*^	0.974^*∗*^	0.975^*∗*^	0.860	0.926^*∗*^	0.952^*∗*^
Total carotenoids	0.949^*∗*^	0.973^*∗*^	0.974^*∗*^	0.857	0.924^*∗*^	0.950^*∗*^
Condensed tannins	0.952^*∗*^	0.975^*∗*^	0.976 ^*∗*^	0.861	0.927^*∗*^	0.952^*∗*^
Hydrolysable tannins	0.948^*∗*^	0.972^*∗*^	0.973 ^*∗*^	0.855	0.923^*∗*^	0.950^*∗*^

^*∗*^ *P* < 0.05. Pearson's correlation coefficient was calculated using Student's *t*-test for all variables at 5% significance levels. 1,1-Diphenyl-2-picrylhydrazyl (DPPH^*∙*^), 2,2′-azino-bis(3-ethylbenzothiazoline-6-sulphonic acid (ABTS^*∙*+^), reducing potential (Fe^3+^/Fe^2^), reactive substances to thiobarbituric acid [TBARS levels induced by 2,2′-azo-bis (2-methylpropionamidine]) dihydrochloride, AAPH), and nitrite content (induced by sodium nitroprusside).

## Data Availability

The data used to support the findings of this study are available from the corresponding author upon request.
